# Biological dosiomic features for the prediction of radiation pneumonitis in esophageal cancer patients

**DOI:** 10.1186/s13014-021-01950-y

**Published:** 2021-11-14

**Authors:** Chanon Puttanawarut, Nat Sirirutbunkajorn, Suphalak Khachonkham, Poompis Pattaranutaporn, Yodchanan Wongsawat

**Affiliations:** 1 Chakri Naruebodindra Medical Institute, Faculty of Medicine Ramathibodi Hospital, Mahidol University, Nakhorn Pathom, Samutprakarn Thailand; 2 Brain-Computer Interface Laboratory, Department of Biomedical Engineering, Faculty of Engineering, Mahidol University, Nakhorn Pathom, Thailand; 3 Department of Diagnostic and Therapeutic Radiology, Faculty of Medicine, Ramathibodi Hospital, Mahidol University, Bangkok, Thailand

**Keywords:** Radiotherapy, Dosiomic, Machine learning, Radiation pneumonitis, Esophageal cancer, Biological dose

## Abstract

**Objective:**

The purpose of this study was to develop a model using dose volume histogram (DVH) and dosiomic features to predict the risk of radiation pneumonitis (RP) in the treatment of esophageal cancer with radiation therapy and to compare the performance of DVH and dosiomic features after adjustment for the effect of fractionation by correcting the dose to the equivalent dose in 2 Gy (EQD2).

**Materials and methods:**

DVH features and dosiomic features were extracted from the 3D dose distribution of 101 esophageal cancer patients. The features were extracted with and without correction to EQD2. A predictive model was trained to predict RP grade ≥ 1 by logistic regression with L1 norm regularization. The models were then evaluated by the areas under the receiver operating characteristic curves (AUCs).

**Result:**

The AUCs of both DVH-based models with and without correction of the dose to EQD2 were 0.66 and 0.66, respectively. Both dosiomic-based models with correction of the dose to EQD2 (AUC = 0.70) and without correction of the dose to EQD2 (AUC = 0.71) showed significant improvement in performance when compared to both DVH-based models. There were no significant differences in the performance of the model by correcting the dose to EQD2.

**Conclusion:**

Dosiomic features can improve the performance of the predictive model for RP compared with that obtained with the DVH-based model.

**Supplementary Information:**

The online version contains supplementary material available at 10.1186/s13014-021-01950-y.

## Introduction

Esophageal cancer is a thoracic cancer for which radiotherapy (RT) is an effective treatment [1]. Radiation pneumonitis (RP) is one of the side effects of thoracic radiation therapy, occurring in patients whose lungs have been irradiated during treatments for malignancy. RP usually develops in the first 6 months after irradiation. Depending on its severity, the symptoms of RP include dyspnea, nonproductive cough and hypoxemia requiring supplemental oxygen. Careful consideration of dose to the lung must be made to limit the occurence of RP which affect radiation planning.

Machine learning (ML) models can be developed as normal tissue complication probability (NTCP) models to predict toxicity arising from radiation. Dosimetric factors extracted from DVH and patient factors such as comorbidity, age, and history of chemotherapy are commonly used as features in predictive models for RP after treatment [2–4]. Recently, texture analysis (TA) approaches, such as radiomics, have emerged that can extract latent information from medical images and improve the performance of predictive models in the field of radiation oncology. TA has also been applied to dose distribution in radiotherapy, referred to as dosiomics. Several researchers have reported improved toxicity prediction performance after radiation therapy by dosiomics [5–7], including for RP [8–10]. However, studies on dosiomics as features for the prediction of RP were performed in lung cancer patients. Due to differences in dose distribution to the lung and other confounding factors in lung cancer that are associated with the risk of RP, such as tumor location [11] and changes in gross tumor volume (GTV) during treatment, a model developed for patients lung cancer may not be applicable to esophageal cancer patients.

Fraction size is an important factor in radiation therapy. The impact of fraction size can be illustrated using the well-known linear quadratic (LQ) model. Fraction size has been shown to be a significant factor of RP [12–14]. However, previous studies using dosiomic features for the prediction of RP did not consider the effect of fraction size.

The purpose of this study was to develop a model with DVH and dosiomic features to predict the risk of RP in esophageal cancer patients treated with radiation therapy and to compare the performance of DVH and dosiomic features with or without consideration of the fraction size effect by correcting the dose distribution to the equivalent dose in 2 Gy (EQD2).

## Material and methods

### Data

The 3D dose distribution of all delivered fractions in 333 esophageal cancer patients > 15 years of age treated with radiation therapy from 201**1** to 2019 was extracted from the Varian Eclipse treatment planning system (TPS) at the Ramathibodi Hospital at Mahidol University. All dose distributions were calculated by Analytical Anisotropic Algorithm (AAA) on Eclipse TPS using free-breathing CT image. This retrospective study was approved by the ethical committee of the Ramathibodi Hospital at Mahidol University. The esophageal cancer patients included in this study were all locally advanced esophageal cancer treated with radiation except for metastatic disease. Patients with a follow-up time under 1 year, a previous history of thoracic radiation therapy, a diagnosis of interstitial lung disease, absence of treatment data and lung metastasis within 1 year were excluded from the study, leaving a total of 101 patients remaining for the analysis. Radiation therapy was delivered with 3D conformal, intensity-modulated radiation therapy (IMRT), volumetric-modulated arc therapy (VMAT) and combined techniques with free breathing. Image guided radiation therapy was done by cone beam CT for the first three fraction and weekly after with daily kilovoltage imaging. The prescription dose ranged from 30 to 60 Gy with 1.8–3 Gy per fraction. A summary of the clinical and treatment characteristics is provided in Table 1 and Additional file 1: Table S3. The code for the extraction of the treatment plan from Varian Eclipse TPS based on the Eclipse Scripting Application Programming Interface (ESAPI) is available at https://github.com/44REAM/ExportFractionDose.git.

**Table 1:** Patient clinical and treatment characteristics

Clinical and treatment characteristic	Median (range)/n (%)
Age	61 (26–93)
Sex	
Male	89 (88%)
Female	12 (12%)
Smoking history	
No	29 (29%)
Active smoking	25 (25%)
Quit smoking < 10 years	33 (33%)
Quit smoking ≥ 10 years	14 (13%)
Stage	
1	4 (4%)
2	3 (3%)
3	71 (70%)
4	23 (23%)
Treatment setting	
CCRT	95 (94%)
RT	6 (6%)
RT aim	
Preoperative	47 (47%)
Postoperative (adjuvant)	1 (1%)
Definitive	49 (48%)
Palliative	4 (4%)
Prescription dose	50.4 (30.0–60.0)
Prescription dose per fraction	1.8 (1.8–3.0)
RT modality	
3D conformal RT	78 (77%)
IMRT/VMAT	9 (9%)
Combine	14 (14%)
RP grade	
0	38 (38%)
1	58 (57%)
2	5 (5%)
3	0 (0%)
4	0 (0%)

RP was reviewed and graded by radiation oncologists based on the National Cancer Institute Common Terminology Criteria for Adverse Events version 5.0 (CTCAE v5.0). RP requiring intubation was deemed to be grade 4. RP requiring oxygen and steroids was deemed to be grade 3. RP requiring steroids or with symptoms that interfered with daily activities was deemed to be grade 2. RP with symptoms or radiographic features without the need for steroids was deemed to be grade 1. RP grades equal to or greater than 1 are labeled positive, and grade 0 is labeled negative for prediction.

### Equivalent dose in 2 Gy fractions

Dose distributions were extracted in fractions. The dose distribution of fraction $\user2{i }$ is referred to as ${\varvec{d}}_{{\varvec{i}}}$ (dose per fraction per voxel). The equivalent dose in the 2 Gy fraction (EQD2) was calculated as follows: [15]

$$EQD2 = \mathop \sum \limits_{{\varvec{i}}} \frac{{d_{i} + d_{i}^{2} /\left( {\alpha /\beta } \right)}}{{1 + 2/\left( {\alpha /\beta } \right)}}$$

The value of the α/β ratio in the equation was assumed to be 3 [16–21]. The equation above is suitable for our dataset because of its compatibility with different doses per fraction per voxel ($d_{i}$). Although we use a similar prescription fraction size (1.8–3 Gy per fraction), the actual dose the patient received in different locations and fractions might be different. For example, the first fraction may have been delivered by an antero-posterior beam, and the second fraction may have been delivered by 2 lateral beams, resulting in different doses per fraction for different voxels. The distribution of the number of beams were showed in Additional file 1: Tables S4, S5 and Figs. S9, S10.

### Features

All dose distributions were resampled to 1.5 × 1.5 × 1.5 mm^3^. DVH features were mean lung dose (MLD), generalized equivalent uniform dose (gEUD), the relative volume of the lung that received a dose greater than x Gy, Vx, ranging from V5 to V70 in 5 Gy steps. Dosiomic features were extracted from the dose distribution using the Pyradiomics library in Python [22]. gEUD was calculate by ${\varvec{gEUD}} = \left( {\mathop \sum \limits_{{\varvec{i}}} {\varvec{D}}_{{\varvec{i}}}^{{\frac{1}{{\varvec{n}}}}} } \right)^{{\varvec{n}}} \user2{ }$. Parameter n was set to 0.99 as previously described [8, 23]. Dosiomic features included in this study were based on the following: first-order statistics (18 features) and texture features (75 features). Prior to extraction of texture features, gray-level intensity was binned to the 100 Gy level with a fixed bin size of 1 Gy. Texture features were based on the gray level cooccurrence matrix (GLCM) (24 features), gray level run length matrix (GLRLM) (16 features), gray level size zone matrix (GLSZM) (16 features), neighborhood gray tone difference matrix (NGTDM) (5 features) and gray level dependence matrix (GLDM) (14 features). Both DVH and dosiomics features were extracted from lung region of interest (ROIs) from either dose distribution with or without correction to EQD2. All features were normalized to the range of 0–1. In summary, 30 DVH features and 186 dosiomic features were extracted from each patient. All analyzed features were show in Additional file 1: Table S1.

### Model building

**Fig. 1:**
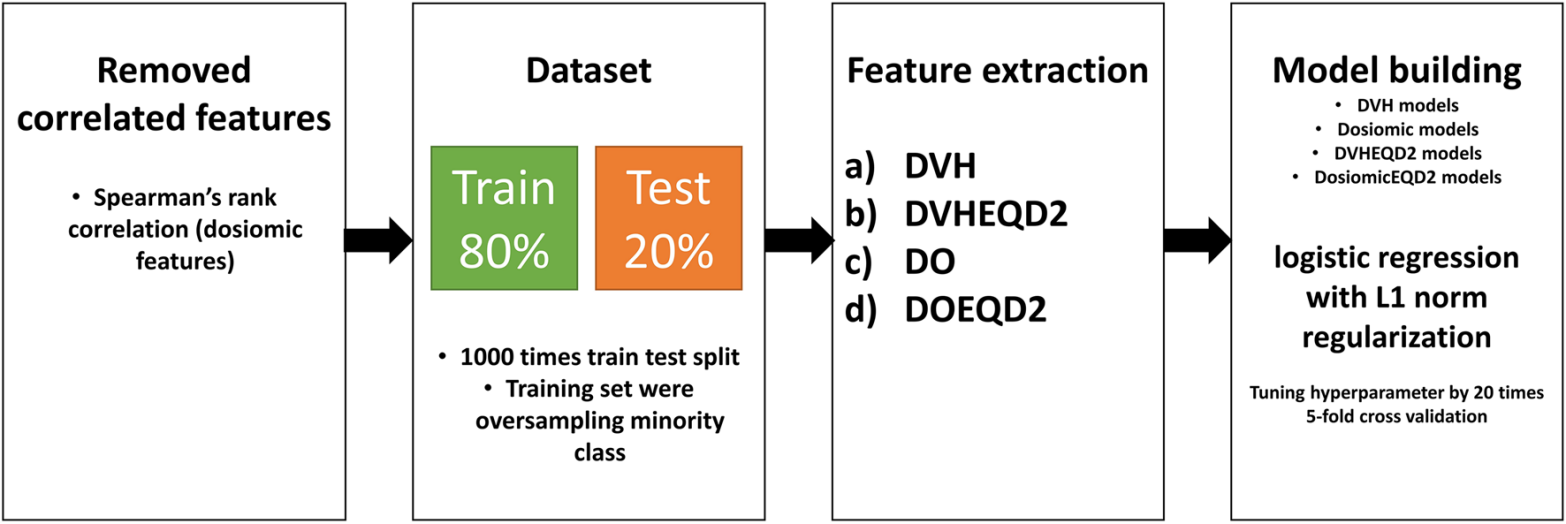
Overview of the process of this study. *DVH* dose volume histogram features, *DO* dosiomic features, *DVHEQD2* DVH features with dose corrected to EQD2, *DOEQD2* dosiomic feature with dose corrected to EQD2

Models were built from feature types as follows: (a) model with DVH features (DVH), (b) model with DVH features corrected to EQD2 dose distribution (DVHEQD2), (c) model with dosiomic features (DO), and (d) model with dosiomic features corrected to EQD2 dose distribution (DOEQD2). First, redundant features were removed only from the dosiomic-based features by Spearman’s rank correlation test to prevent overfitting, which removed other features with high correlation, leaving only one feature [8, 9, 24]. A high correlation coefficient (CC) in Spearman's rank correlation test was defined as CC > 0.8. Before insertion into the model, the data were randomly separated into 1000 instances of the training set (80%) and test set (20%). The minority class in the training set was randomly oversampled with replacement to equalize the two classes. Multivariate logistic regression with L1 norm regularization (LASSO) was used [25–27]. LASSO was performed to prevent overfitting and can also be used as embedded feature selection in the logistic regression model by shrinking the coefficient of unimportant features to zero. The regularized strength of the L1 norm was determined by 20 rounds of inner loop fivefold cross validation to maximize the average area under the curve (AUC) of the receiver operating characteristic curve (ROC) on the training set using grid search. An overview of the process is demonstrated in Fig. 1. Further details regarding the model building and hyperparameter tuning are presented in Additional file 1: Supplementary S1.

### Evaluation

The mean, standard deviation (SD) and 10th–90th percentile of AUC in the test set of 1000 models in each group were calculated. A Z-test was used to test the statistical significance of the AUC between the 4 models. Statistical analyses were performed using the Python and SciPy packages [28]. A *p* value < 0.05 was considered significant.

## Results

The distribution of RP grade was showed in Additional file 1: Table S3. Due to the RT modality can affect the texture of dose distribution, the association between RT modality and RP for RP grade 0 and grade ≥ 1 was test by cri-square. The result showed that there are no association between RT modality and RP (*p* value 0.89). This result was consistent to previous study which no benefits of IMRT over 3D conformal technique in reduction of RP [29].

In the model building process, dosiomic features were progressively eliminated due to the high correlation among features. The remaining number of features were 20 and 24 in the DO and DOEQD2, respectively, and these features were used to the train logistic regression model (Additional file 1: Table S2).

The mean of the ROC curve is provided in Additional file 1: Fig. S1. The AUCs of the models with DVH, DVHEQD2, DO and DOEQD2 were 0.66 ± 0.11, 0.66 ± 0.11, 0.70 ± 0.11 and 0.71 ± 0.11, respectively (Table 2). The data showed that there was no significant difference observed in the performance when correcting dose distribution to EQD2 in either model (DVH model and DVHEQD2 model (*p* value = 0.56), DO model and DOEQD2 model (*p* value = 0.73)). Both dosiomic models performed better than the dosimetric-based models (*p* value < 0.01).

**Table 2:** Logistic regression model results

	OR	10th, 90th OR	AUC	10th, 90th AUC
DVH models			0.66 ± 1.1	0.52, 0.80
V40	5.58	0, 10.77		
V45	2.16	0, 8.89		
DVHEQD2 models			0.66 ± 0.11	0.51, 0.80
V40	4.90	0, 12.10		
V35	1.34	0, 9.30		
DO models			0.70 ± 0.11	0.55, 0.85
NTGDM busyness	− 0.07	− 0.10, − 0.04		
90 percentile	0.01	0, 0.02		
GLRLM LongRunGrayLevelEmphasis	0.51^a^	0, 2.05^a^		
DOEQD2 models			0.71 ± 0.11	0.57, 0.84
NTGDM busyness	− 0.07	− 0.11, − 0.04		
90 percentile	0.02	0, 0.11		
GLSZM LowGrayLevelZoneEmphasis	25.96	− 117.66, 0		

Features corresponding to the model showed only features that were selected more than 50% of the time. OR was adjusted to the actual value of features (not normalized value)

^a^Reported normalized value due to very low OR

**Fig. 2:**
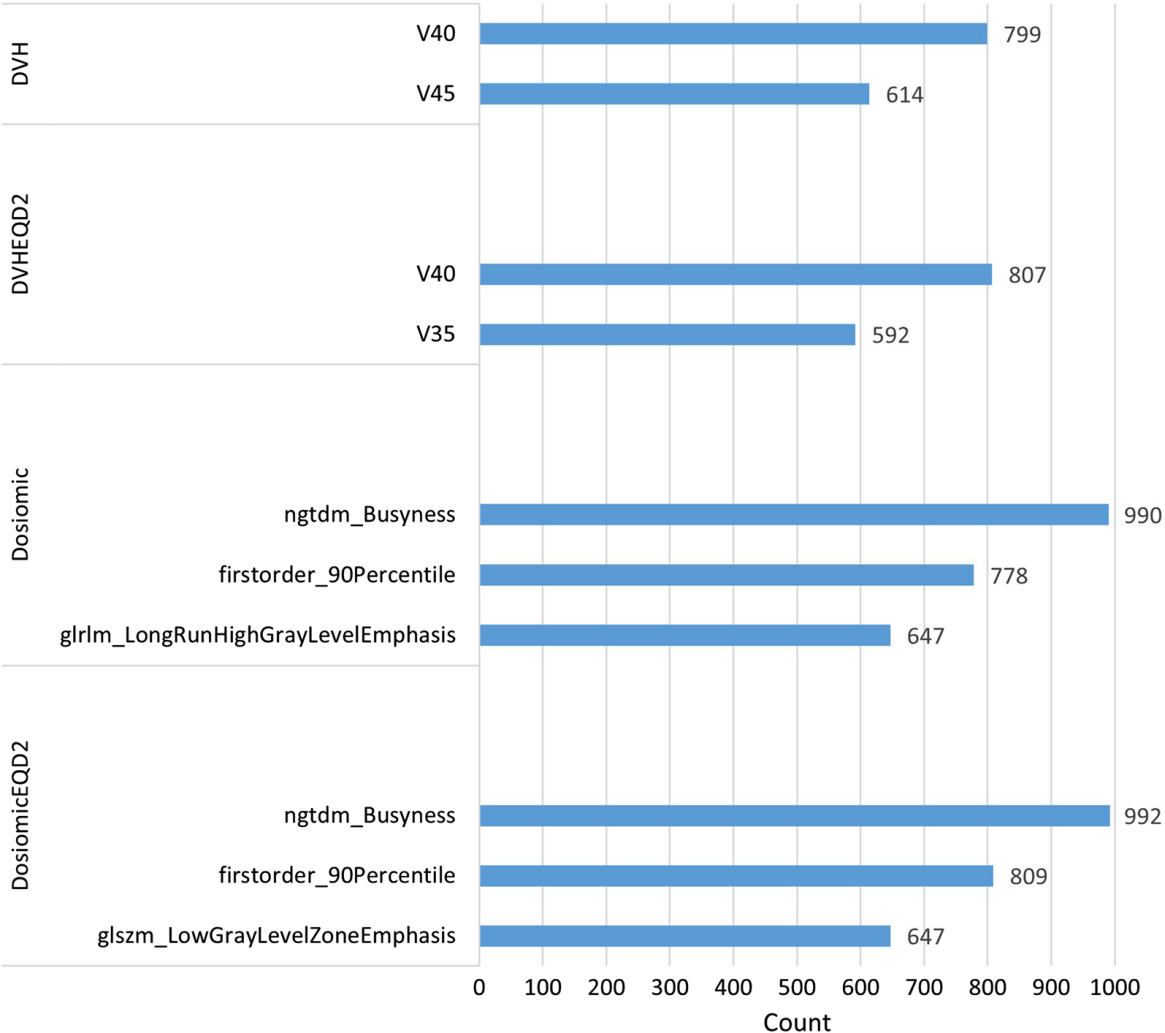
Most selected features from 1000 models. Only features that were selected more than 500 times are shown (50%)

Features that were selected in more than 50% of all groups are shown in Fig. 2. The median odds ratios (ORs) with 10th–90th ORs of the features that were selected in more than 50% of the groups are shown in Table 2. Features that were selected in more than 50% in the DVH and DVHEQD2 groups were V40 and V**45** and V35 and V40, respectively. Busyness from NTGDM and the 90th percentile were selected in the dosiomic-based group. Other features that were selected for more than 50% in DO were LongRunHighGrayLevelEmphasis (LRHGLE) from GLRLM and LowGrayLevelZoneEmphasis (LGLZE) from GLSZM in DOEQD2. We further investigated the correlation between features with and without correction to EQD2 of the selected features. The correlations among the most selected features in DVH, DVHEQD2, DO and DOEQD2 are shown in Additional file 1: Figs. S2, S3, S4, S5, S6, S7 and S8. Most of the selected features had a high CC (CC ≥ 0.9) between features with and without correction to EQD2. Only LGLZE had a CC of 0.73.

## Discussion

To the best of our knowledge, our study is the first in dosiomics to analyze radiation delivered per fraction instead of the sum of all fractions and to investigate the effect of EQD2 on DVH and dosiomic features. In other studies, the dose per fraction per voxel of the predictive model of IMRT, VMAT and stereotactic body radiation therapy (SBRT) was usually the same for all fractions. If the fraction size is the same for all fractions, the total dose can simply be divided by the number of fractions to obtain the fraction size per voxel. However, our data include 3D conformal techniques that can involve several beam arrangements and treatment phases for different fractions depending on the clinical judgment of the radiation oncologist.

In the DVH and DVHEQD2 models, the most selected features were V35, V40 and V45. The difference in selected features between DVH and DVHEQD2 can be explained by correction of the dose to EQD2, resulting in a lower dose. The overall values of the features are shown in Additional file 1: Figs. S2, S3 and S4. Previous studies usually indicate the importance of V20 [30–34] and MLD [30, 32, 33, 35–37] as predictors of RP. However, our study revealed that more in than 50% of the reads, V35, V40 and V45 (Fig. 2) were selected. The results of a previous study of patients treated with CCRT with the 3D conformal technique suggested that V30 is a predictive factor for RP grade ≥ 2 [31] in lung cancer patients. Although V30 was not among the most selected features in our study, this might be because the grade of RP was different, and in [31], Vx other than V20 and V30 were not investigated. Another study also revealed V40 to be an important factor, but the end point was severe RP (RP ≥ 3) [33] in lung cancer patients. In studies of esophageal cancer patients, V30 has been shown to be significantly correlated with acute RP (RP occurs within 3 months post radiation therapy) with 3D conformal techniques [34]. These researchers investigated V50 but did not find a significant correlation with RP; however, V35, V40 and V45 were not investigated. In another study of esophageal cancer patients treated with CCRT by 3D conformal technique, V5–V60 were analyzed and none of these factors were found to be associated with RP grade ≥ 2 [38].

In our study, the two most selected dosiomic features in the DO and DOEQD2 groups were Busyness in the NTGDM and the 90th percentile of dose (Fig. 2). The third most selected feature in DO and DOEQD2 was different (LRHGLE in DO and LGLZE in DOEQD2). This was because in DOEQD2, LRHGLE was eliminated due to its high correlation with other features, while LRHGLE in DO was not eliminated (Additional file 1: Table S1). Two prior studies in dosiomic features and RP reported the most important dosiomic features in GLCM group. Contrast from GLCM was found to be the most predictive feature of RP grade ≥ 2 in non-small cell lung cancer (NSCLC) treated with VMAT [8]. Correlation with GLCM extracted from wavelet transformation of dose imaging in lung regions with a dose greater than 5 Gy was found to be a significant feature in a predictive model of RP grade ≥ 2 in early-stage NSCLC treated with SBRT [9]. Another study in dosiomics were analyzed reported the use of a combination of 8 features in the GLSZM group and 1 feature in the GLCM group, which resulted in the best performance for the prediction of late RP (RP grade ≥ 2 developed 6 months after the start of RT) in lung cancer treated with VMAT. However, we did not find any feature that was selected for more than 50% in the GLCM group and found one only feature in the GLSZM group. These results are different from those of previous studies (Fig. 2). This might be due to the difference in the nature of the dose distribution and disease of the patients studied, since in our dataset, most of the patients were treated by the 3D conformal technique, and the study was performed in esophageal cancer patients.

The most selected feature in the dosiomic-based model was busyness. Busyness indicates the spatial frequency of intensity changes [39]. In dose distribution, busyness can be interpreted as an intensity change in radiation dose. From our result, busyness is negatively correlated with RP. Thus, a low spatial frequency change in radiation dose intensity is correlated with RP. The second most selected feature in the dosiomic-based model is the 90th percentile. By estimating parameters from the Lyman–Kutcher–Burman (LKB) model, the lungs have usually been interpreted as parallel organs [13, 23]. In parallel organs, the 90th percentile of the dose (one “hot” spot) in the dose distribution might not be a good feature. However, in this study, we predicted RP grade 1, which may manifest only in the form of local changes in radiographic images. Therefore, the lungs can be viewed as a series organ in this case.

Previous studies demonstrated that the use of dosiomic features can improve the performance of predictive models in RP [8–10]. In our study, dosiomic features also showed significant improvement from dosimetric features. We also further investigated the performance of features with and without correction to EQD2. The results showed that no significant improvement from DVH to DVHEQD2 and DO to DOEQD2 could be found. Previous research studies of biological dose accumulation and conventional accumulation dose showed that the difference between biological dose accumulation and conventional accumulation dose increases depending on daily variation of dose in theoretical prediction [15]. Their results indicated that the biological dose would be unlikely to affect the dose–response model, which was based on DVH in standard fractionation. In other words, this indicates there is no impact on the outcome whether we accumulate the dose by biological effect or not. Their results might explain why our DVH and DVHEQD2 models were not different. For dosiomic features, our results did not show a significant difference in performance between DO and DOEQD2. However, the results showed differences in the most selected features (Fig. 2). By looking at the CC of the selected features between DO and DOEQD2, some dosiomic features (LGLZE) had lower a CC. This may lead to different selected features between DO and DOEQD2. Although we did not find any difference in performance between the DO and DOEQD2 models, we recommend correction of dose to biological dose due to potential differences in the selected features. Since the cell survival from the LQ model is nonlinear, correcting the dose to EQD2 (or other biological doses) could improve validity over using dose with linear summation. As previous studies reported a correlation between fraction size and RP [12–14], we can extract more information about dose distribution for predicting RP if we correct the dose distribution first.

We believe that our data reflect the population of esophageal cancer patients since patient characteristic (age, sex and tumor stage) were similar to other study of esophageal cancer [27, 29, 37]. The incident of RP grade ≥ 2 in esophageal cancer was report by systematic review about 6.6% which similar to our study (5%), although information for grade 1 RP were lacking. Patients with history of interstitial lung disease (ILD) in our dataset were excluded because it was difficult to differentiate between ILD and radiation pneumonitis (RP). The treatment of ILD and RP are also similar (prescription of steroid) which can lead to misclassification. The majority of patient in our dataset were treated with 3D conformal technique which may limit generalization to other RT technique (e.g. IMRT). For patient with dose distribution and consistent number of beams throughout all fraction, the result of the predictive model should not much be difference since the performance of dosiomic model were the same with or without correction of dose distribution to EQD2.

The limitation of this study was the paucity of RP events requiring steroids (5 patients), which corresponded to RP grade ≥ 2. Therefore, the prediction in this study was performed primarily on RP grade ≥ 1, which might limit its usefulness in actual clinical practice, though the model of RP grade ≥ 1 can be used for ruling out RP grade ≥ 2 as well which could aid in some clinical decision. In a previous study, it was suggested that the impact of biological dose on the DVH parameter may be higher with the hypofraction regimen [15]. The second limitation was that in our study, most data were of standard fraction size. The effect of biological dose with the hypofraction regimen on dosiomic features should be investigated.

## Conclusion

In this study, we demonstrated the potential of dosiomic features that can improve the performance of a predictive model of radiation pneumonitis grade ≥ 1 in esophageal cancer patients in comparison with that obtained with a DVH-based model. We also confirmed the benefit of dosiomic features in the prediction of RP in 3D conformal RT. Correction of dose per fraction to EQD2 did not improve the performance of the predictive model in standard fractionation. However, we recommend correcting the dose to the biological dose for dose-related analysis. Although this model may not be useful in clinical situations due to the limitation of training data, the insight gained may prove useful in further endeavors to develop a predictive model of RP for use in radiation treatment planning.

## Supplementary Information

**Additional file 1**. **Table S1:** All features analysed in this study. **Table S2:** Remaining dosiomic features before input to ML model. **Table S3:** Radiation pneumonitis grade categorized by RT modality. **Table S4:** Distribution of the number of beams by RP grade. **Figure S1:** ROC of DVH, DVHEQD2, DO and DOEQD2. **Figure S2:** Scatter plot between DVH and DVHEQD2 of V35. **Figure S3:** Scatter plot between DVH and DVHEQD2 of V40. **Figure S4:** Scatter plot between DVH and DVHEQD2 of V45. **Figure S5:** Scatter plot between DO and DOEQD2 of Busyness from NGTDM. **Figure S6:** Scatter plot between DO and DOEQD2 of 90th percentile. **Figure S7:** Scatter plot between DO and DOEQD2 of LGLZE from GLSZM. **Figure S8:** Scatter plot between DO and DOEQD2 of LRHGLE from GLRLM. **Figure S9:** distribution of number of beams by RP grade in 3D conformal technique.

## Data Availability

The datasets in this study are not publicly available. The code for extraction of data from TPS was available publicly at https://github.com/44REAM/ExportFractionDose.git.

## Abbreviations

DVH: Dose-volume histogram
EQD2: Equivalent dose in 2 Gy
DVHEQD2: Dose-volume histogram calculate from dose distribution with EQD2
DO: Dosiomic
DOEQD2: Dosiomic calculate from dose distribution with EQD2
RP: Radiation pneumonitis
RT: Radiation therapy
ROC: Receiver operating characteristic
AUCs: Area under the curve
ML: Machine learning
NTCP: Normal tissue complication probability
TA: Texture analysis
GTV: Gross tumor volume
LQ: Linear quadratic
TPS: Treatment planning system
IMRT: Intensity-modulated radiation therapy
VMAT: Volumetric-modulated arc therapy
SBRT: Stereotactic body radiation therapy
CTCAE: National Cancer Institute Common Terminology Criteria for Adverse Events
ESAPI: Eclipse Scripting Application Programming Interface
gEUD: Generalized equivalent uniform dose
Vx: The relative volume of the lung that received a dose greater than x Gy
CC: Correlation coefficient
ROI: Region of interest
GLCM: Gray level cooccurrence matrix
GLRLM: Gray level run length matrix
GLSZM: Gray level size zone matrix
NGTDM: Neighborhood gray tone difference matrix
GLDM: Gray level dependence matrix
SD: Standard deviation
LRHGLE: “LongRunHighGrayLevelEmphasis”
LGLZE: “LowGrayLevelZoneEmphasis”
LKB: Lyman–Kutcher–Burman
CT: Computed tomography

## References

[1] Tu C-C, Hsu P-K (2018). The frontline of esophageal cancer treatment: questions to be asked and answered. Ann Transl Med.

[2] Hope AJ, Lindsay PE, El Naqa I, Alaly JR, Vicic M, Bradley JD (2006). Modeling radiation pneumonitis risk with clinical, dosimetric, and spatial parameters. Int J Radiat Oncol Biol Phys.

[3] Valdes G, Solberg TD, Heskel M, Ungar L, Simone CB (2018). Using machine learning to predict radiation pneumonitis in patients with stage I non-small cell lung cancer treated with stereotactic body radiation therapy. Physiol Behav.

[4] Yakar M, Etiz D, Metintas M, Ak G, Celik O (2021). Prediction of radiation pneumonitis with machine learning in stage III lung cancer: a pilot study. Technol Cancer Res Treat.

[5] Rossi L, Bijman R, Schillemans W, Aluwini S, Cavedon C, Witte M (2018). Texture analysis of 3D dose distributions for predictive modelling of toxicity rates in radiotherapy. Radiother Oncol.

[6] Wu A, Li Y, Qi M, Lu X, Jia Q, Guo F (2020). Dosiomics improves prediction of locoregional recurrence for intensity modulated radiotherapy treated head and neck cancer cases. Oral Oncol.

[7] Gabryś HS, Buettner F, Sterzing F, Hauswald H, Bangert M (2018). Design and selection of machine learning methods using radiomics and dosiomics for normal tissue complication probability modeling of xerostomia. Front Oncol.

[8] Liang B, Yan H, Tian Y, Chen X, Yan L, Zhang T (2019). Dosiomics: extracting 3D spatial features from dose distribution to predict incidence of radiation pneumonitis. Front Oncol.

[9] Adachi T, Nakamura M, Shintani T, Mitsuyoshi T, Kakino R, Ogata T (2021). Multi-institutional dose-segmented dosiomic analysis for predicting radiation pneumonitis after lung stereotactic body radiation therapy. Med Phys.

[10] Bourbonne V, Da-ano R, Jaouen V, Lucia F, Dissaux G, Bert J (2021). Radiomics analysis of 3D dose distributions to predict toxicity of radiotherapy for lung cancer. Radiother Oncol.

[11] Li F, Liu H, Wu H, Liang S, Xu Y (2021). Risk factors for radiation pneumonitis in lung cancer patients with subclinical interstitial lung disease after thoracic radiation therapy. Radiat Oncol.

[12] Mack B, Iii R, Gandara DR, Yuo H, Swift PS, Kroll S (1995). Radiation pneumonitis following combined modality therapy for lung cancer: analysis of prognostic factors. J Clin Oncol.

[13] Marks LB, Bentzen SM, Deasy JO, Kong FM, Bradley JD, Vogelius IS (2010). Radiation dose-volume effects in the lung. Int J Radiat Oncol Biol Phys.

[14] Shank B, Chu FCH, Dinsmore R, Kapoor N, Kirkpatrick D, Teitelbaum H (1983). Hyperfractionated total body irradiation for bone marrow transplantation. Results in seventy leukemia patients with allogeneic transplants. Int J Radiat Oncol Biol Phys.

[15] Niebuhr NI, Splinter M, Bostel T, Seco J, Hentschke CM, Floca RO (2021). Biologically consistent dose accumulation using daily patient imaging. Radiat Oncol.

[16] Borst GR, Ishikawa M, Nijkamp J, Hauptmann M, Shirato H, Bengua G (2010). Radiation pneumonitis after hypofractionated radiotherapy: evaluation of the LQ(L) model and different dose parameters. Int J Radiat Oncol Biol Phys.

[17] Kwa SLS, Lebesque JV, Theuws JCM, Marks LB, Munley MT, Bentel G (1970). Radiation pneumonitis as a function of mean lung dose: an analysis of pooled data of 540 patients. N Engl J Med.

[18] Ren C, Ji T, Liu T, Dang J, Li G (2018). The risk and predictors for severe radiation pneumonitis in lung cancer patients treated with thoracic reirradiation. Radiat Oncol.

[19] Dhami G, Zeng J, Vesselle HJ, Kinahan PE, Miyaoka RS, Patel SA (2018). Framework for radiation pneumonitis risk stratification based on anatomic and perfused lung dosimetry. Physiol Behav.

[20] Selvaray J, Lebesque JV, Hope A, Guckenberger M, Werner-Wasik M, Peulen H (2019). Modeling radiation pneumonitis of pulmonary stereotactic body radiotherapy: the impact of a local dose–effect relationship for lung perfusion loss. Radiother Oncol.

[21] Thor M, Deasy J, Iyer A, Bendau E, Fontanella A, Apte A (2016). Toward personalized dose-prescription in locally advanced non- small cell lung cancer: validation of published normal tissue complication probability models. Physiol Behav.

[22] Van Griethuysen JJM, Fedorov A, Parmar C, Hosny A, Aucoin N, Narayan V (2017). Computational radiomics system to decode the radiographic phenotype. Cancer Res.

[23] Seppenwoolde Y, Lebesque JV, De Jaeger K, Belderbos JSA, Boersma LJ, Schilstra C (2003). Comparing different NTCP models that predict the incidence of radiation pneumonitis. Int J Radiat Oncol Biol Phys.

[24] Kakino R, Nakamura M, Mitsuyoshi T, Shintani T, Kokubo M, Negoro Y (2020). Application and limitation of radiomics approach to prognostic prediction for lung stereotactic body radiotherapy using breath-hold CT images with random survival forest: a multi-institutional study. Med Phys.

[25] Schaake W, van der Schaaf A, van Dijk LV, Bongaerts AHH, van den Bergh ACM, Langendijk JA (2016). Normal tissue complication probability (NTCP) models for late rectal bleeding, stool frequency and fecal incontinence after radiotherapy in prostate cancer NTCP models for anorectal side effects patients. Radiother Oncol.

[26] Krafft SP, Rao A, Stingo F, Briere TM, Court LE, Liao Z (2018). The utility of quantitative CT radiomics features for improved prediction of radiation pneumonitis. Med Phys.

[27] Hirose TA, Arimura H, Ninomiya K, Yoshitake T, Fukunaga JI, Shioyama Y (2020). Radiomic prediction of radiation pneumonitis on pretreatment planning computed tomography images prior to lung cancer stereotactic body radiation therapy. Sci Rep.

[28] Virtanen P, Gommers R, Oliphant TE, Haberland M, Reddy T, Cournapeau D (2020). SciPy 1.0: fundamental algorithms for scientific computing in Python. Nat Methods.

[29] Haefner MF, Lang K, Verma V, Koerber SA, Uhlmann L, Debus J (2017). Intensity-modulated versus 3-dimensional conformal radiotherapy in the definitive treatment of esophageal cancer: comparison of outcomes and acute toxicity. Radiat Oncol.

[30] Ryckman JM, Baine M, Carmicheal J, Osayande F, Sleightholm R, Samson K, et al. Correction to: Correlation of dosimetric factors with the development of symptomatic radiation pneumonitis in stereotactic body radiotherapy (Radiation Oncology, (2020), 15, 1, (33), 10.1186/s13014-020-1479-6). Radiat Oncol 2021;16:1–15. 10.1186/s13014-021-01797-3. 10.1186/s13014-020-1479-6 PMC7020355 32054487

[31] Ramella S, Trodella L, Mineo TC, Pompeo E, Stimato G, Gaudino D (2010). Adding ipsilateral V20 and V30 to conventional dosimetric constraints predicts radiation pneumonitis in stage IIIA-B NSCLC treated with combined-modality therapy. Int J Radiat Oncol Biol Phys.

[32] Luna JM, Chao HH, Diffenderfer ES, Valdes G, Chinniah C, Ma G (2019). Predicting radiation pneumonitis in locally advanced stage II–III non-small cell lung cancer using machine learning. Radiother Oncol.

[33] Kim TH, Cho KH, Pyo HR, Lee JS, Zo JI, Lee DH (2005). Radiology dose-volumetric parameters for predicting severe radiation pneumonitis after three-dimensional conformal radiation therapy for lung. Radiology.

[34] Kumar G, Rawat S, Puri A, Sharma MK, Chadha P, Babu AG (2012). Analysis of dose-volume parameters predicting radiation pneumonitis in patients with esophageal cancer treated with 3D-conformal radiation therapy or IMRT. Jpn J Radiol.

[35] Kocak Z, Borst GR, Zeng J, Zhou S, Hollis DR, Zhang J, Evans ES, Folz RJ, Wong T, Kahn D, Belderbos JS (2011). Prospective assessment of dosimetric/physiologic-based models for predicting radiation pneumonitis. Bone.

[36] Oh D, Ahn YC, Park HC, Lim DH, Han Y (2009). Prediction of radiation pneumonitis following high-dose thoracic radiation therapy by 3 gy/fraction for non-small cell lung cancer: analysis of clinical and dosimetric factors. Jpn J Clin Oncol.

[37] Tonison JJ, Fischer SG, Viehrig M, Welz S, Boeke S, Zwirner K (2019). Radiation pneumonitis after intensity-modulated radiotherapy for esophageal cancer: institutional data and a systematic review. Sci Rep.

[38] Wang S, Liao Z, Wei X, Liu HH, Tucker SL, Hu C (2008). Association between systemic chemotherapy before chemoradiation and increased risk of treatment-related pneumonitis in esophageal cancer patients treated with definitive chemoradiotherapy. J Thorac Oncol.

[39] Amadasun M, King R (1989). Texural features corresponding to texural properties. IEEE Trans Syst Man Cybern.

