# Supplementary material for: Biological dosiomic features for the prediction of radiation pneumonitis in esophageal cancer patients
Source: Radiat Oncol. 2021 Nov 14;16:220. doi: 10.1186/s13014-021-01950-y (PMC8591796; doi:10.1186/s13014-021-01950-y)
Supplement: Supplementary file 1 — Additional file 1. Table S1: All features analysed in this study. Table S2: Remaining dosiomic features before input to ML model. Table S3: Radiation pneumonitis grade categorized by RT modality. Table S4: Distribution of the number of beams by RP grade. Figure S1: ROC of DVH, DVHEQD2, DO and DOEQD2. Figure S2: Scatter plot between DVH and DVHEQD2 of V35. Figure S3: Scatter plot between DVH and DVHEQD2 of V40. Figure S4: Scatter plot between DVH and DVHEQD2 of V45. Figure S5: Scatter plot between DO and DOEQD2 of Busyness from NGTDM. Figure S6: Scatter plot between DO and DOEQD2 of 90th percentile. Figure S7: Scatter plot between DO and DOEQD2 of LGLZE from GLSZM. Figure S8: Scatter plot between DO and DOEQD2 of LRHGLE from GLRLM. Figure S9: distribution of number of beams by RP grade in 3D conformal technique. [file 13014_2021_1950_MOESM1_ESM.docx]

**Supplementary material: Biological dosiomic features for the prediction of radiation pneumonitis in esophageal cancer patients**

**Model building**

First, redundant features were removed only from the dosiomic-based features by Spearman’s rank correlation test to prevent overfitting, which removed other features with high, leaving only one feature. We preserved the feature in group as order from “First order”, ”GLCM”,” GLRLM”, “GLSZM”, “NGTDM” and “GLDM” with lesser alphabetical order in feature name.

The upsampling were done separately in each train-test split using “RandomOverSampler” function in imbalanced learn package in python.

For binary classification objective function of logistic regression model with L1 norm regularization can be written as below:

$$L=\left| \left| w \right| \right|_{1}+ C\sum_{i} \log\left( \exp\left( -y_{i}\left( X_{i}^{T}w+b \right) \right)+1 \right)$$

Where w and b are model coefficients, $X_{i}$ and $y_{i}$ is input features and input class for patient $i$ respectively. Parameter C is hyperparameter of L1 norm regularization. Strength of regularization is proportional to 1/C. The hyperparameter of logistic regression with L1 norm regularization model was tune by grid search for C $\in$ [0.05, 0.1, 0.5, 1] by selecting C that maximize mean AUC of 20 times 5-fold cross validation.

Models were train in Python using Sklearn library. Other hyperparameter in model are optimizer, which call “solver” in Sklearn, was set to “liblinear” and maximum iteration, which call “max_iter” was set to 10000, the remaining hyperparameter are set to default.

**Table**

**Table S1: All features analysed in this study.**

|  | Features |
| --- | --- |
| DVH features | Mean lung dose (MLD), generalized equivalent uniform dose (gEUD), V5 – V70 |
| First Order (18 features) | **10th percentile, 90th percentile, Energy, Entropy, Interquartile Range, Kurtosis, Maximum, Absolute Deviation (MAD), Mean, Median, Minimum, Range, Robust Mean Absolute Deviation (rMAD), Root Mean Squared (RMS), Skewness, Total Energy, Uniformity, Variance** |
| Gray Level Co-occurrence Matrix (GLCM) (24 features) | **Autocorrelation, Joint Average, Cluster Prominence, Cluster Shade, Cluster Tendency, Contrast, Correlation, Difference Average, Difference Entropy, Difference Variance, Joint Energy, Joint Entropy, Informational Measure of Correlation (IMC), Informational Measure of Correlation (IMC) 2, Inverse Difference Moment (IDM), Maximal Correlation Coefficient (MCC), Inverse Difference Moment Normalized (IDMN), Inverse Difference (ID), Inverse Difference Normalized (IDN), Inverse Variance, Maximum Probability, Sum Average, Sum Entropy, Sum of Squares** |
| Gray Level Run Length Matrix (GLRLM) (16 features) | **Short Run Emphasis (SRE), Long Run Emphasis (LRE), Gray Level Non-Uniformity (GLN), Gray Level Non-Uniformity Normalized (GLNN), Run Length Non-Uniformity (RLN), Run Length Non-Uniformity Normalized (RLNN), Run Percentage (RP), Gray Level Variance (GLV), Run Variance (RV), Run Entropy (RE), Low Gray Level Run Emphasis (LGLRE), High Gray Level Run Emphasis (HGLRE), Short Run Low Gray Level Emphasis (SRLGLE), Short Run High Gray Level Emphasis (SRHGLE), Long Run Low Gray Level Emphasis (LRLGLE), Long Run High Gray Level Emphasis (LRHGLE)** |
| Gray Level Size Zone Matrix (GLSZM) (16 features) | **Small Area Emphasis (SAE), Large Area Emphasis (LAE), Gray Level Non-Uniformity (GLN), Gray Level Non-Uniformity Normalized (GLNN), Size-Zone Non-Uniformity (SZN), Size-Zone Non-Uniformity Normalized (SZNN), Zone Percentage (ZP), Gray Level Variance (GLV), Zone Variance (ZV), Zone Entropy (ZE), Low Gray Level Zone Emphasis (LGLZE), High Gray Level Zone Emphasis (HGLZE), Small Area Low Gray Level Emphasis (SALGLE), Small Area High Gray Level Emphasis (SAHGLE), Large Area Low Gray Level Emphasis (LALGLE), Large Area High Gray Level Emphasis (LAHGLE)** |
| Neighbouring Gray Tone Difference Matrix (NGTDM) (5 features) | Coarseness, Contrast, Busyness, Complexity, Strength |
| Gray Level Dependence Matrix (GLDM) (14 features) | **Small Dependence Emphasis (SDE), Large Dependence Emphasis (LDE), Gray Level Non-Uniformity (GLN), Dependence Non-Uniformity (DN), Dependence Non-Uniformity Normalized (DNN), Gray Level Variance (GLV), Dependence Variance (DV), Dependence Entropy (DE), Low Gray Level Emphasis (LGLE), High Gray Level Emphasis (HGLE), Small Dependence Low Gray Level Emphasis (SDLGLE), Small Dependence High Gray Level Emphasis (SDHGLE), Large Dependence Low Gray Level Emphasis (LDLGLE), Large Dependence High Gray Level Emphasis (LDHGLE)** |

**Table S2: Remaining dosiomic features before input to ML model.**

|  | Feature name |
| --- | --- |
| DO (24 features) | firstorder_10Percentile  firstorder_90Percentile  firstorder_Energy  firstorder_Entropy  firstorder_InterquartileRange  firstorder_Kurtosis  firstorder_Maximum  glcm_Correlation  glcm_Imc2  glrlm_GrayLevelNonUniformity  glrlm_GrayLevelNonUniformityNormalized  glrlm_LongRunHighGrayLevelEmphasis  glrlm_RunEntropydosiomic  glszm_GrayLevelNonUniformity  glszm_GrayLevelNonUniformityNormalized  glszm_HighGrayLevelZoneEmphasis glszm_LargeAreaHighGrayLevelEmphasis  glszm_LowGrayLevelZoneEmphasis  glszm_SizeZoneNonUniformityNormalized  glszm_ZoneEntropy  ngtdm_Busyness  ngtdm_Complexity  ngtdm_Strength  gldm_DependenceVariance |
| DOEQD2 (20 features) | firstorder_10Percentile  firstorder_90Percentile  firstorder_Energy  firstorder_Entropy  firstorder_InterquartileRange  firstorder_Maximum  glcm_Correlation  glrlm_GrayLevelNonUniformity glrlm_GrayLevelNonUniformityNormalized glrlm_RunEntropy  glszm_GrayLevelNonUniformity  glszm_GrayLevelNonUniformityNormalized glszm_HighGrayLevelZoneEmphasis  glszm_LargeAreaHighGrayLevelEmphasis  glszm_LowGrayLevelZoneEmphasis  glszm_SizeZoneNonUniformity  glszm_SizeZoneNonUniformityNormalized glszm_ZoneEntropy  ngtdm_Busyness  ngtdm_Strength |

**Table S3: Radiation pneumonitis grade categorized by RT modality**

| **RT modality** | **Grade 0** | **Grade ≥ 1** | **Grade 2** | **Total** |
| --- | --- | --- | --- | --- |
| 3D conformal RT | 29 (37%) | 46 (59%) | 3 (4%) | 78 (100%) |
| IMRT/VMAT | 3 (33%) | 5 (56%) | 1 (11%) | 9 (100%) |
| Combine | 6 (43%) | 7 (50%) | 1 (7%) | 14 (100%) |

**Table S4: Distribution of the number of beams by RP grade**

| Number of beams | **Grade 0** | **Grade 1** | **Grade 2** |
| --- | --- | --- | --- |
| 2 | 1 (33.33%) | 2 (66.67%) | - |
| 3 | 1 (100%) | - | - |
| 4 | 2 (66.67%) | 1 (33.33%) | - |
| 5 | 1 (50.00%) | 1 (50.00%) | - |
| 6 | 5 (62.50%) | 2 (25.00%) | 1 (12.50%) |
| 7 | 5 (41.67%) | 6 (50.00%) | 1 (8.33%) |
| 8 | - | 8 (100%) | - |
| 9 | 1 (20.00%) | 4 (80%) | - |
| 10 | 3 (23.08%) | 10 (76.92%) | - |
| 11 | 3 (42.86%) | 4 (57.14%) | - |
| 12 | 2 (50.00%) | 2 (50.00%) | - |
| 13 | 3 (75.00%) | - | 1 (25.00%) |
| 14 | 1 (50.00%) | 1 (50.00%) | - |
| 15 | - | 2 (100%) | - |
| 16 | 1 (50.00%) | 1 (50.00%) | - |
| 19 | - | 2 (100.00%) | - |

**Figure**


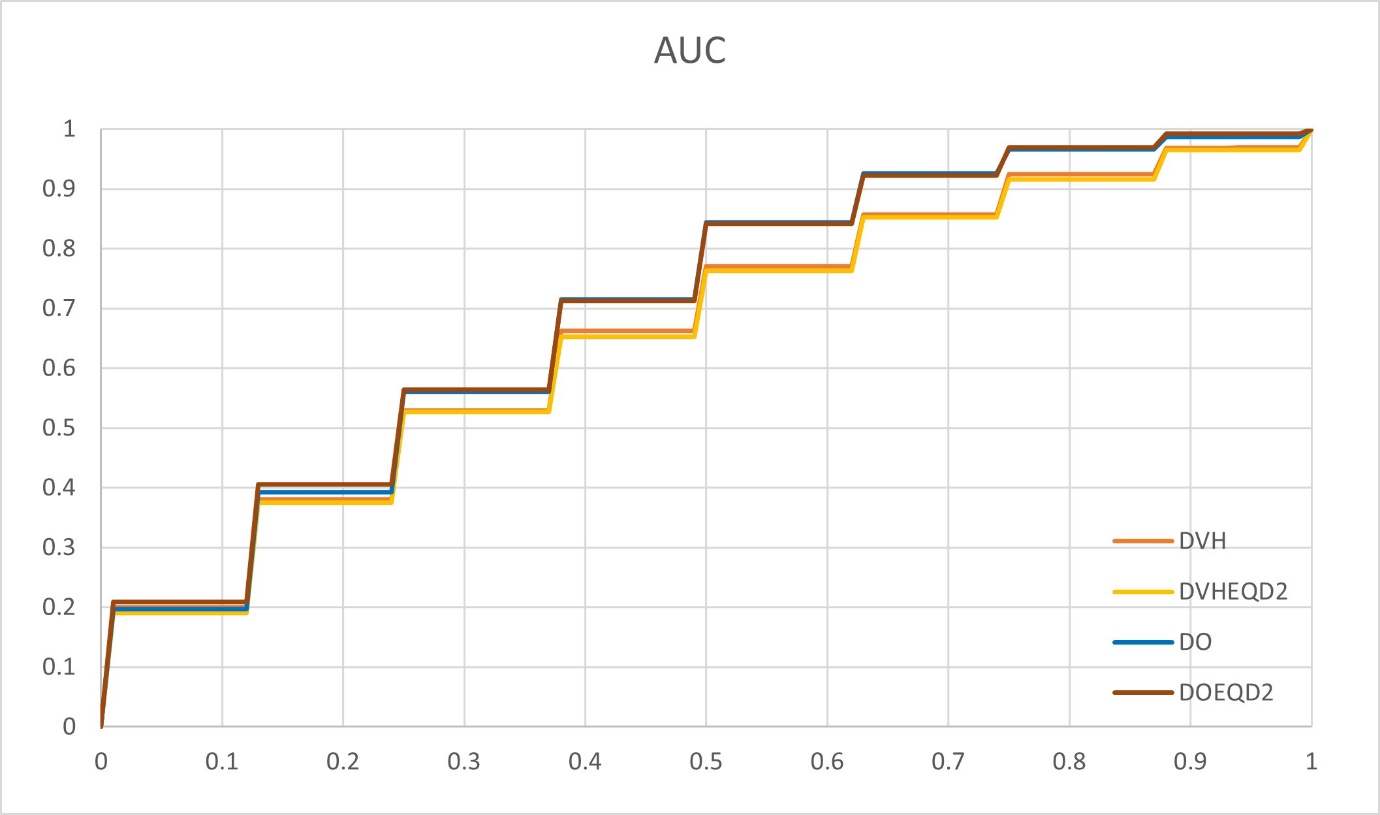


**Figure S1: ROC of DVH, DVHEQD2, DO and DOEQD2**


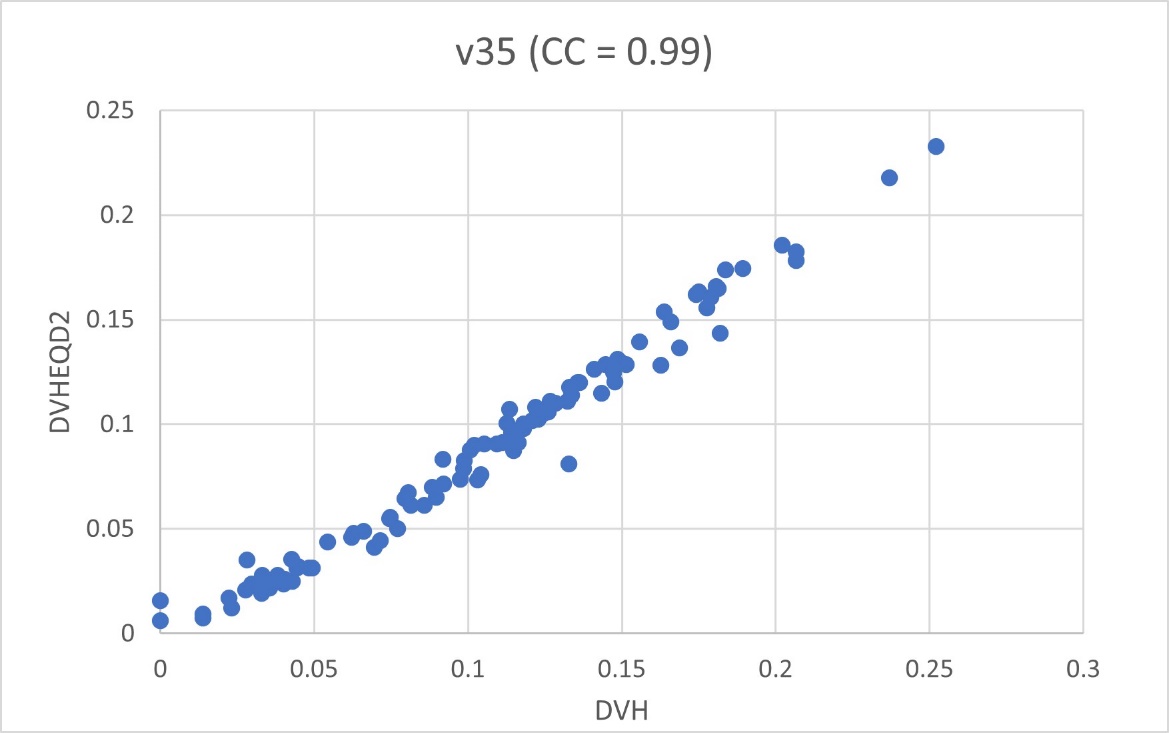


**Figure S2: Scatter plot between DVH and DVHEQD2 of V35**


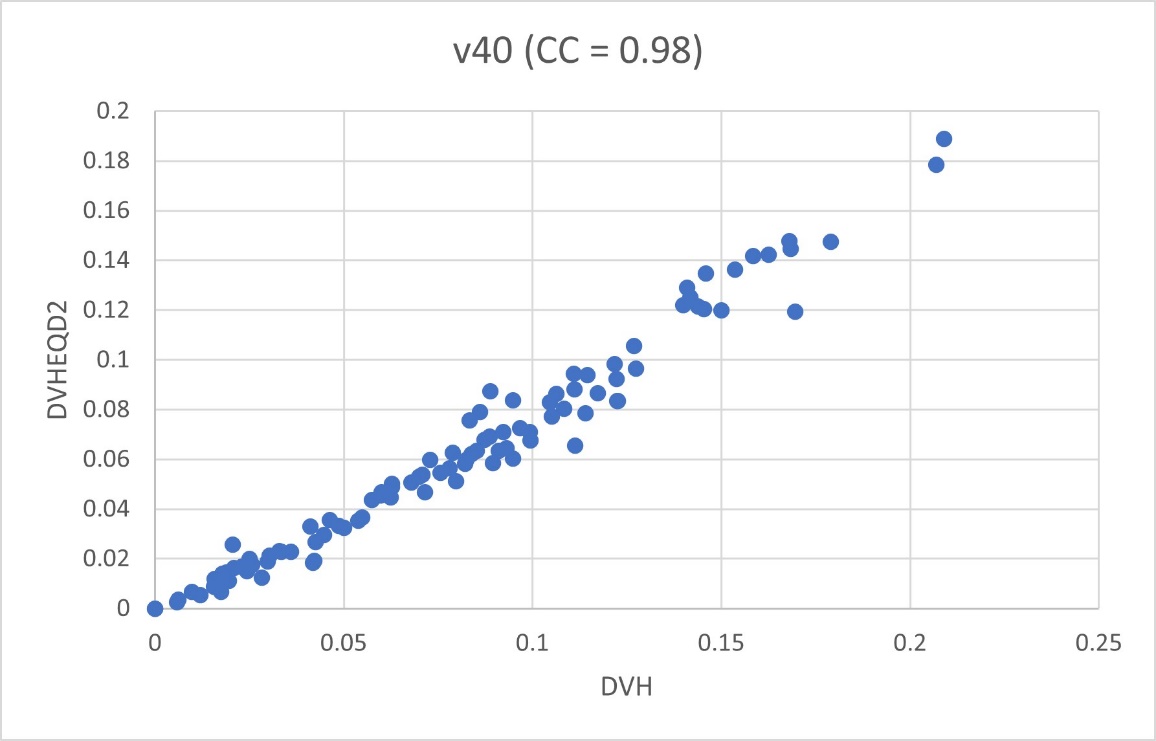

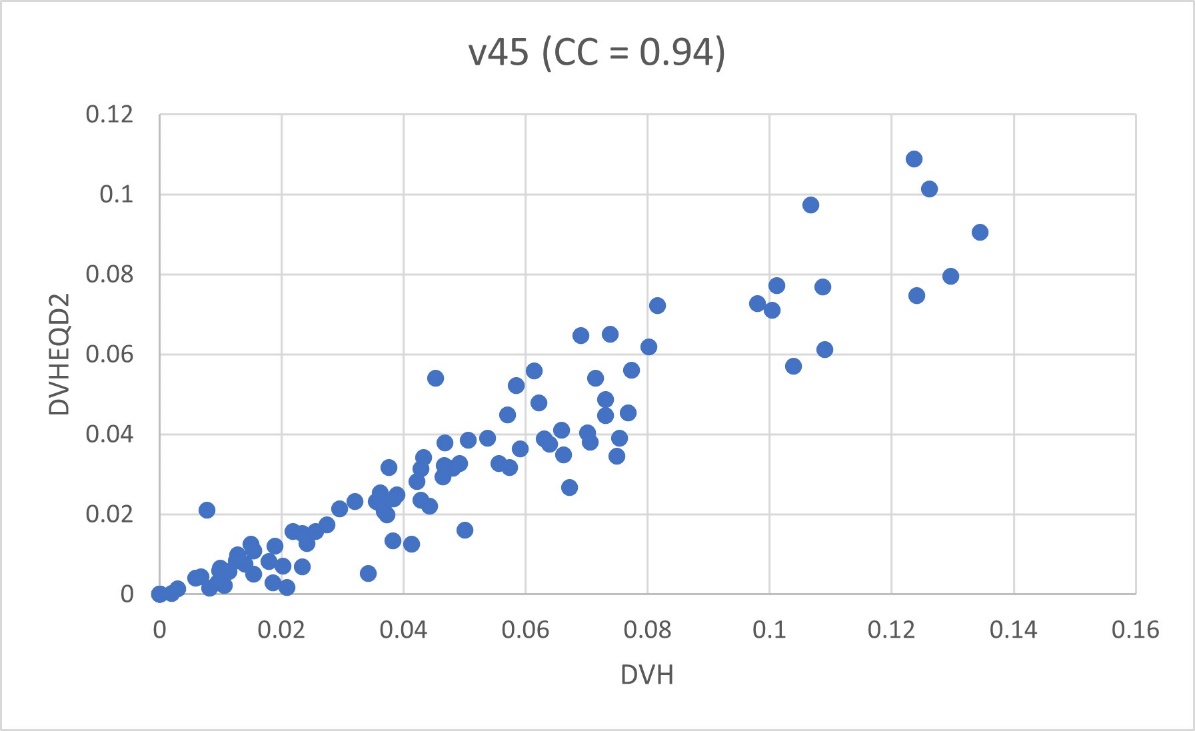

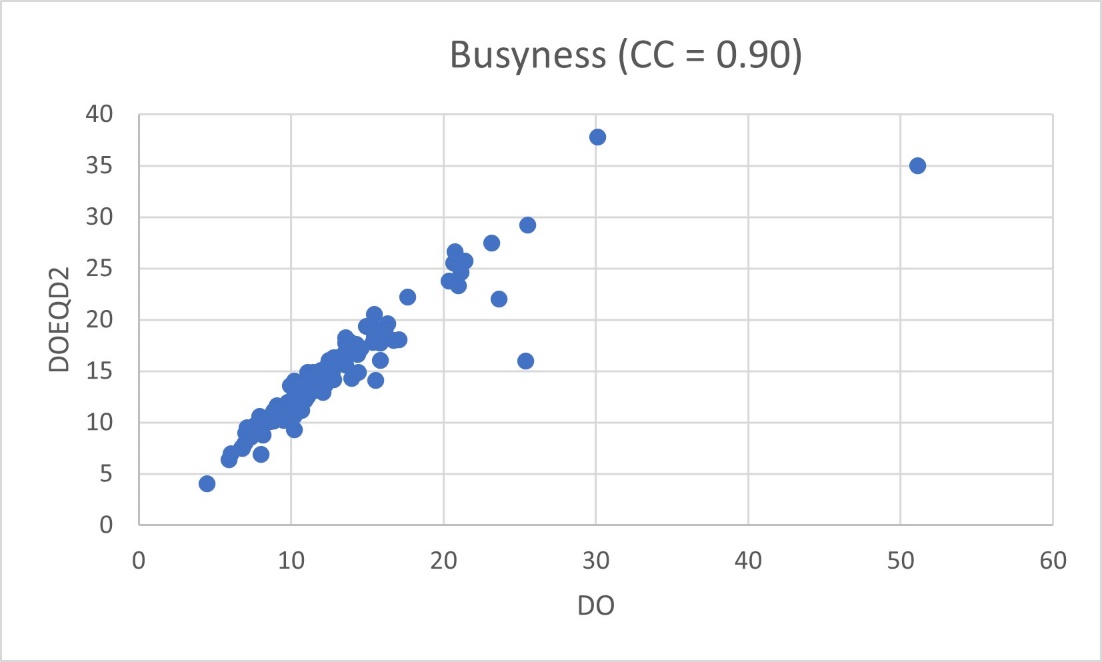


**Figure S3: Scatter plot between DVH and DVHEQD2 of V40**

**Figure S4: Scatter plot between DVH and DVHEQD2 of V45**

**Figure S5: Scatter plot between DO and DOEQD2 of Busyness from NGTDM**

***
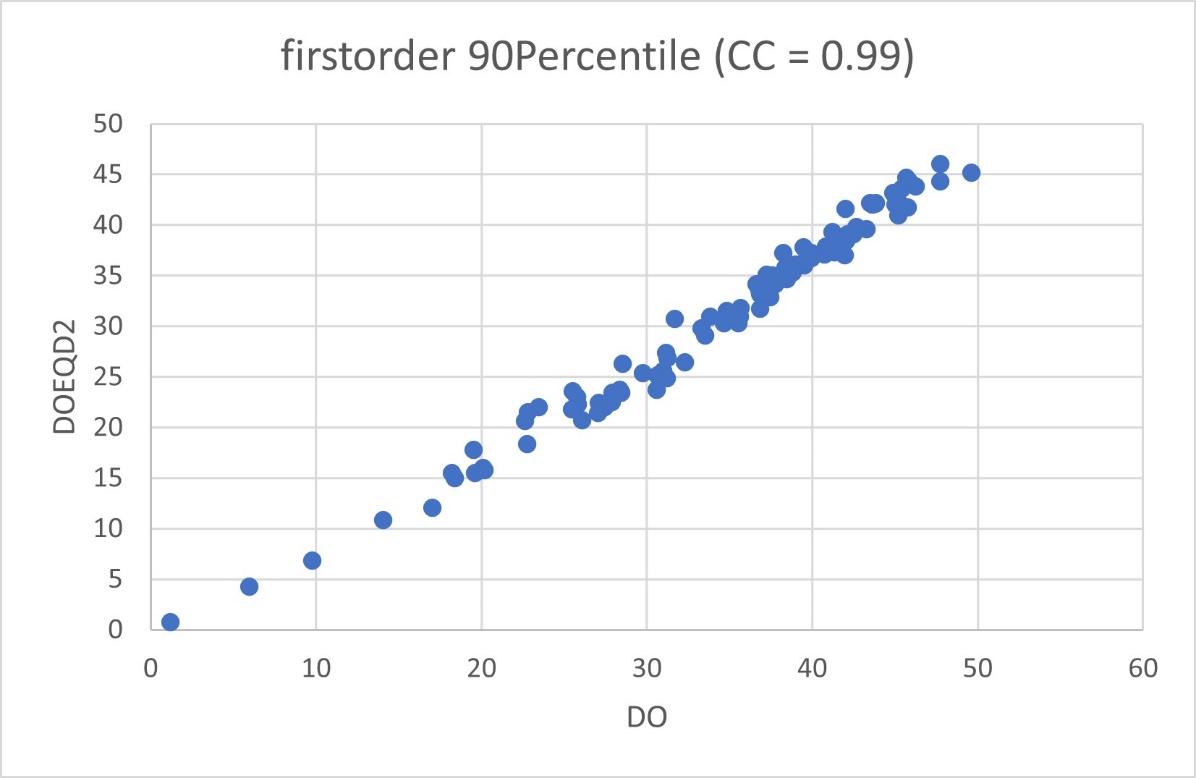
***

**Figure S6: Scatter plot between DO and DOEQD2 of 90^th^ percentile**

**
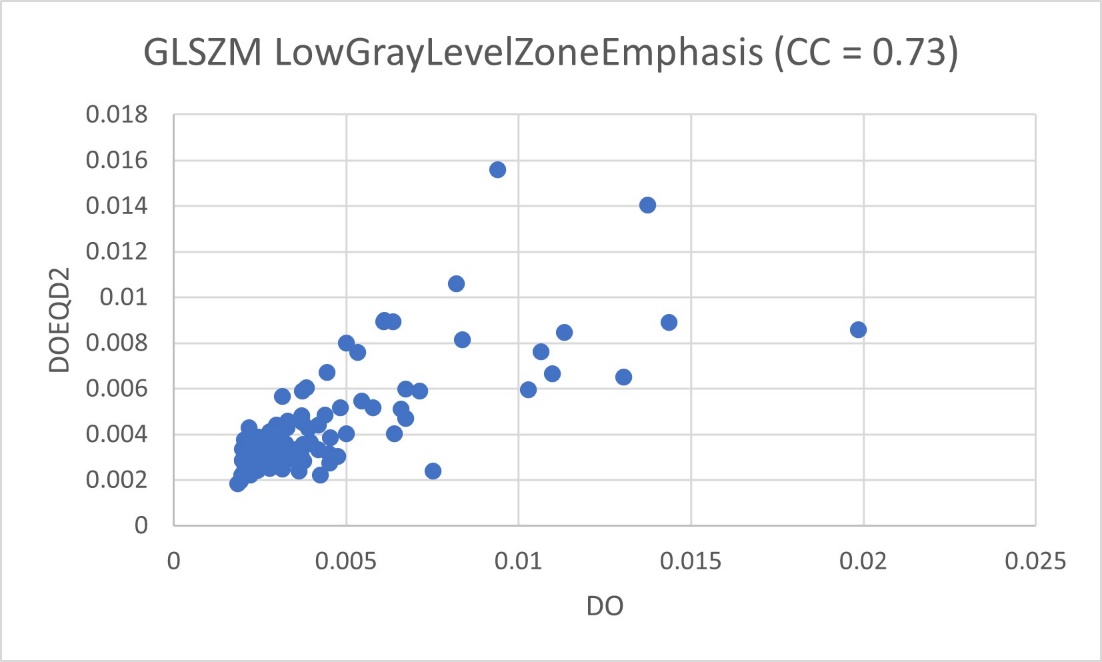
**

**Figure S7: Scatter plot between DO and DOEQD2 of LGLZE from GLSZM**

**
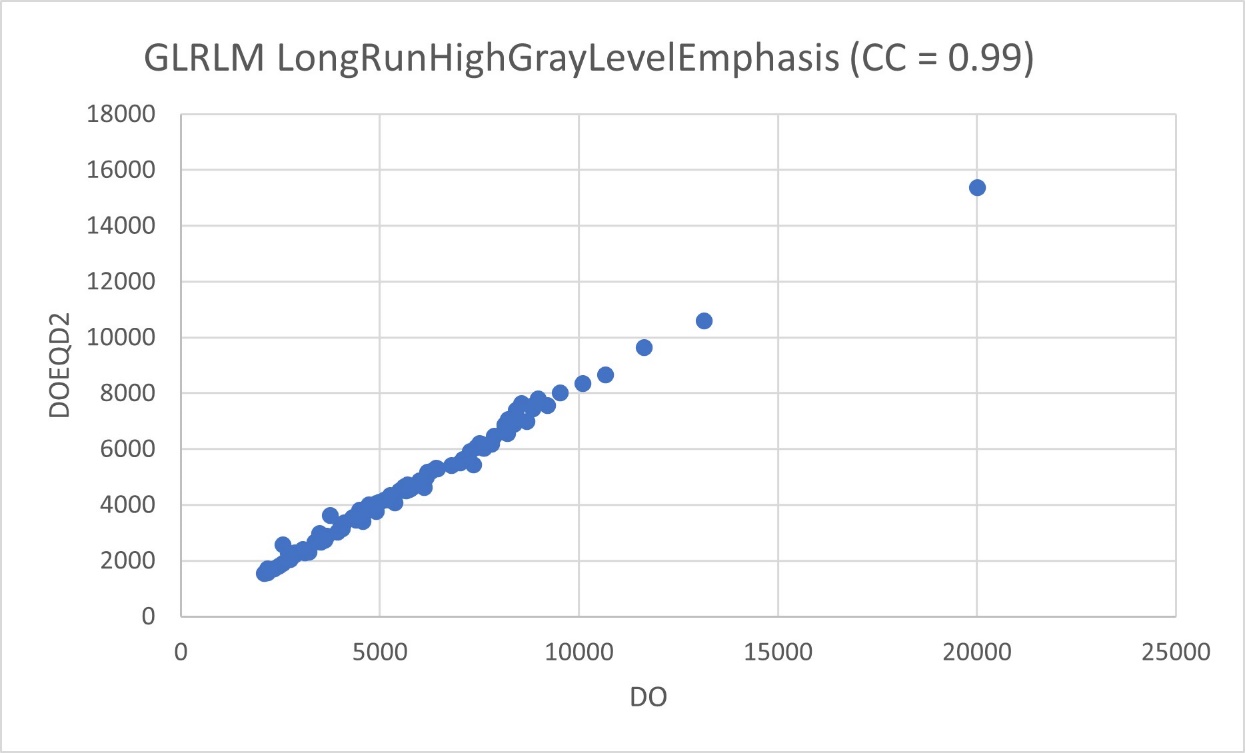
**

**Figure S8: Scatter plot between DO and DOEQD2 of LRHGLE from GLRLM**

**Figure S9: distribution of number of beams by RP grade in 3D conformal technique**
